# Re-situations of scientific knowledge: a case study of a skirmish over clusters vs clines in human population genomics

**DOI:** 10.1007/s40656-022-00497-9

**Published:** 2022-04-21

**Authors:** James Griesemer, Carlos Andrés Barragán

**Affiliations:** 1 Department of Philosophy, University of California, Davis, One Shields Avenue, Davis, CA 95616 USA; 2 Department of Science and Technology Studies, University of California, Davis, One Shields Avenue, Davis, CA 95616 USA

**Keywords:** Re-situation, Scientific knowledge, Human population genomics, Ancestry studies, Skirmish, Objects of knowledge

## Abstract

We track and analyze the re-situation of scientific knowledge in the field of human population genomics ancestry studies. We understand re-situation as a process of accommodating the direct or indirect transfer of objects of knowledge from one site/situation to (one or many) other sites/situations. Our take on the concept borrows from Mary S. Morgan’s work on facts traveling while expanding it to include other objects of knowledge such as models, data, software, findings, and visualizations. We structure a specific case study by tracking the re-situation of these objects between three research projects studying human population diversity reported in three articles in *Science*, *Genome Research* and *PLoS Genetics* between 2002 and 2005. We characterize these three engagements as a unit of analysis, a “skirmish,” in order to compare: (a) the divergence of interests in how life-scientists answer similar research questions and (b) to track the challenging transformation of workflows in research laboratories as these scientific objects are re-situated individually or in bundles. Our analysis of the case study shows that an accurate understanding of re-situation requires tracking the whole bundle of objects in a project because they interact in particular key ways. The absence or dismissal of these interactions opens the door to unforeseen trade-offs, misunderstandings and misrepresentations about research design(s) and workflow(s) and what these say about the questions asked and the findings produced.

## Introduction to the phenomenon of “re-situation” of scientific knowledge

In scientific work, it is often less than obvious whether two projects address the same question with subtly different approaches, or address subtly different questions with similar approaches. Either way, different answers from similar projects may result. It is thus also less than obvious how to understand when “an” answer to “a” question becomes a rival to a “different” answer to “the” question. This problem becomes all the more pressing when two projects are linked because one is framed as “responding” to the other, particularly if “objects of knowledge” used or generated in the originating project become “re-situated,” that is pushed or pulled, into the responding project and used there.^1^ Such re-situations raise questions of appropriateness to the new circumstances and the status of the response in so far as it depends on the project it challenges.

Here, we call the resources and tools that are used by such projects “objects of knowledge.” This provides us with a generic term for the sorts of “things” that might be “re-situated” when one team builds a project that relates to another. Examples of such objects we consider include models, data, software findings, and visualizations. Re-situation is a term we borrow from Morgan (2014) but use to describe the travel of more than facts^2^ (Howlett & Morgan, 2011; Leonelli, 2011) or data (Leonelli & Tempini, 2020). Settings we consider in a case study include research programs, projects, teams, and local work settings.^3^ What we characterize as “situations” are problems arising in settings that must be resolved in order to get research work done. Sometimes these problems are the research questions scientists seek to articulate and answer, but other times the problems are challenges to methodology, practice, or procedure faced by re-situation of scientific knowledge (from now on RSK) into different settings. In the latter kinds of cases, re-situation disrupts or complicates established workflows, repertoires, know-how, or even community-wide institutions or common knowledge needed to produce or evaluate scientific knowledge, that is, to answer research questions.^4^ The sites or settings into which objects of knowledge might be re-situated could be located in the same or a different laboratory, the same or a different project, the same or a different context of application, or even beyond the research field of concern altogether. We are interested in how workflows, objects of knowledge, contexts, and narratives change due to these “re-situations.”^5^

### The case study

In this article, we report on an exchange of views in the published literature of a field we call human population genomics ancestry studies (HPGA). We call it “ancestry studies” in part to distinguish this kind of work from biomedical or forensic applications of population genomics resources and tools to human subjects.^6^ We call the field HPGA because there is no single term in the literature referring to these studies over the historical span of our larger project, roughly the 1980s to the present, with precursors extending as early as the 1960s.^7^ In this case study of RSK, there was an *originating project* (Rosenberg et al., 2002), a *challenge project* (Serre & Pääbo, 2004) working with similar models, data, software, and findings but questioning the interpretation of these and other objects of knowledge in the originating project, followed in turn by a *response project* (Rosenberg et al., 2005) addressing the challenge project and going beyond it in some respects.^8^

These three papers frame a particular form of (historical and contemporary) engagement, a “skirmish,” in which the minimal unit of analysis is an original site of production of the object(s) of scientific knowledge being re-situated, a challenge situation, and a response situation.^9^ Still more complex skirmishes are of course possible and in the context of many historical and scientific debates and controversies, plausible. Any published exchange of views can seem to go dormant and then be taken up again much later, so there may be larger units of analysis for the skirmish we discuss. Indeed, the exchange of views might instead continue, but be transformed and diversify in various ways, so that it becomes hard for analysts to trace it either historically or across contemporaneous scientific literatures. This would become challenging, even for analysts versed in some aspects of the technical literature of the field, because the controversy might continue into quite different realms of science, e.g., of statistics or computer science. Thus we acknowledge, in presenting this case study, that our findings may not prove to be very robust if other publications engaging our focal three papers turn out to be important for the phenomena we seek to elucidate. Despite this potential limitation on our findings, the strategy of analyzing re-situations as we propose may nevertheless be fruitful in recognizing and studying those larger contexts in which skirmishes as we characterize them here occur.

Noah A. Rosenberg (at the time at the University of Southern California, USC) and six coauthors (from the University of Chicago, the Marshfield Medical Research Foundation in Wisconsin, the Centre d’Étude du Polymorphisme Humain (CEPH) of the Fondation Jean Dausset in Paris, Yale University, the Vavilov Institute of General Genetics in Moscow, and Stanford University) published their paper in a leading general journal, *Science* (Rosenberg et al., 2002). This was part of a *research program* to explore the genetic structure of human populations: the Human Genome Diversity Project (HGDP; see Cavalli-Sforza et al., 1991; Cann et al., 2002; Cavalli-Sforza, 2005).^10^ The analysis relied on a dataset of 377 autosomal microsatellite loci (short tandem repeats, STR) in 1056 individual subjects from 52 globally sampled populations.^11^ The analysis also relied on a population genetic model which assumed ancestral humans migrated “out-of-Africa” and thus that allele frequencies in the sub-divided human population would be correlated across local populations due to their shared ancestry (Cann et al., 1987; Cavalli-Sforza et al., 1994; Rosenberg et al., 2005). Within local populations, the model assumed allele frequencies would typically be in Hardy–Weinberg equilibrium, so differences in (selectively neutral) allele frequencies would be explained by migration and/or genetic drift.

The research team generated data clusters using a software package, STRUCTURE, developed by some of the co-authors, together with others in an overlapping team: Pritchard et al. (2000). The main finding was that, without using any information about the population cultural identities of the individual samples (“meta-data” to the genomic data), the genotype information clustered into groupings of samples based on similar patterns of allele presence/absence that strongly correlate with continent-scale regions. In other words, in this dataset (derived from global, five-continent sampling) genotyping suggested the existence of five major population “clusters.”^12^

The flow of work of the Rosenberg et al. (2002) project began with the design of the study through consideration of the availability of samples collected and maintained by the CEPH.^13^ Samples from a subset of cell lines, modified from Marshfield screening set 10, were selected to represent a world-wide sample and genotyped by the Mammalian Genotyping Service (see Rosenberg et al., 2002, Supplement).^14^ After modifying the dataset from the HGDP-CEPH Cell Line Panel, the generated data had to be prepared (“cleaned”) so as to be analyzable via the procedures of the software package STRUCTURE (Pritchard et al., 2000). Then, the data were analyzed and software-generated output reported in tables of numbers. The results were also visualized in stacked bar charts showing each of the 1056 individuals as a line partitioned into a number of colored segments representing the individuals’ “estimated membership fractions in K clusters” (Rosenberg et al., 2002, Figure 1, legend, p. 2382). The findings about the genetic structure of the 52 human populations sampled are reported in terms of percent of variance within and among various levels of population structure (world, continent, or regions within continent) and in terms of the patterns in the bar chart visualizations. We discuss these “clusters” in Sect. 2.

Our focus on Rosenberg et al. (2002) and the project behind it is not scientific knowledge production per se, but rather what happens to its objects of knowledge when they are “re-situated” from one setting or context to another, e.g., from the workflow where an object is produced to another workflow where it is further used or “consumed.” The “other” site could be in the same or a different laboratory, the same or a different project, the same or a different context of application (e.g., HPGA, HPGB, HPGE, or HPGF, or even beyond human population genomics, see Footnote 8). We are interested in how workflows, objects of knowledge, contexts, and narratives change due to these “re-situations.” For this reason, our minimal unit of analysis must be larger than a single publication such as Rosenberg et al. (2002). To be interpretable as a skirmish, our unit should be smaller but also different than a whole research program such as the HGDP or the research trajectory of a whole laboratory like Noah Rosenberg’s, Svante Pääbo’s or Jonathan K. Pritchard’s. The work in a whole field on a topic, or all of the work characterizable as HPGA, or the topic as covered within even a single organization, such as at Stanford University or the Max Planck Institute for Evolutionary Anthropology, or of the field over a period of its history, are also units too broad for our present purposes. Rather, our minimum unit here is a series of publications reporting studies linked by their use or re-use of one or more objects of knowledge. In our larger project we will eventually need to take into consideration the laboratories, research teams, and organizations, and networks conducting the research and producing those publications in a wider consideration of RSK phenomena (Griesemer & Barragán, 2018).^15^ Again, the most close-focused kind of re-situation that concerns us, as an aid to articulating concepts, is when knowledge moves from a producer scientific laboratory or team to another laboratory or team, or even more “locally,” as when re-deployed by the same laboratory or team in a study subsequent to the one that produced it, within the same or a related “project.”

Because we aim to track knowledge in terms of the kinds of objects mentioned before (and a few others), studying re-situation in any given instance becomes a complex job.^16^ Did the model in Rosenberg et al. (2002) get re-deployed to analyze quite different (and maybe inappropriate) datasets? Did the dataset, already only a subset of the HGDP data available from CEPH, get reused by other groups without the models, software or findings used or generated by the original producers or recombined with other data to form new datasets? Did the re-situated dataset or finding get re-examined using a software package organized and operated on rather different analytical assumptions or computational principles than the original analysis? Does similarity (or difference) in findings between the original production and the re-situated ones reflect robustness (or fragility) of the original findings or of the “methods” (or workflows) to differences between the original and re-situated contexts? Or, do similarities in objects of knowledge in different projects reflect pseudo-robustness due to compensating “errors” in these locally crafted complexes of components put together in different local circumstances? Or, are they due to the introduction of still other objects of knowledge untracked as they moved into the re-situated workflow, constituting “hidden variables” in the complex dynamics of research that travels beyond the boundaries of a local context of production?

Here, we seek to understand a very local re-situation phenomenon manifested in a skirmish between the team that published Rosenberg et al. (2002) and a team challenging their data, models, and findings by Serre and Pääbo (2004), and a response by Rosenberg et al. (2005) deploying enhanced datasets, considering alternative models, reexamining the original and challenge findings, and using an enhanced version of the software.

The Rosenberg et al. (2002) paper was widely noticed, studied, evaluated, and criticized in the literature of HPGA studies and beyond, including in popular media (e.g., Wade, 2002, 2014). As of this writing, the paper has been cited over 3000 times.^17^ The paper stirred controversy about all four of the kinds of objects of knowledge mentioned: the population genetic *model* to generate and interpret the findings, the *dataset* used (along with the sampling procedure to generate it), the *software* to analyze it, and the *findings*, large and small, reported from it. Some life-scientists, social critics, and popular media reporters expressed concern that the paper reinforces a genetic or biological conception of human races because it reported finding continent-scale genetic differences which can be interpreted as corresponding to some traditional conceptions of race (e.g., Wade, 2002, 2014; Coop et al., 2014; Feldman, 2014; see also Wills, 2017).

This reading was contrary to the authors’ own avoidance of any mention of race, in favor of a discussion of “ancestry.”^18^ Another dimension of their larger *finding* was that most human genetic variation is among individuals within populations, that a much smaller amount is among populations within regions or continents, and only a few percent of genotypic differences is among continents. This finding was in line with previous findings and arguments (e.g., Lewontin, 1972), and argued against interpreting patterns of genetic structure among human populations as support for reductionist, biological concepts of race.^19^ However, questions about the models, dataset, software, and findings have led to a variety of critiques, challenges, and parallel or divergent projects. Among these, a prominent concern has been around how findings in Rosenberg et al. (2002) could be read or not as “biologizing” race, both by life and social scientists (e.g., Bolnick, 2008; Foster & Sharp, 2004; Gannett, 2005; Glasgow, 2003; Marks, 2010; REGWG, 2005; Royal & Dunston, 2004). Yet for the purpose of the analysis at hand (the skirmish) we cannot focus on the numerous and highly informative insights this scholarship has produced.^20^ Rather, we want here to track and understand fairly localized scientific practices in terms of workflows of production and re-situation that mobilize multiple kinds of objects of knowledge: what disrupts them, enhances them, or leads to invention of entirely new ones or even new kinds, and how.

### Re-situation of scientific knowledge or just a “deidealization” step?

The emerging philosophical (and historiographic) literature on RSK focuses on what happens when a model, as a type of scientific object, is re-situated (Cartwright, 2012; Cartwright & Hardie, 2012; Morgan, 2014), i.e., made relevant in new locations by fitting the model into a different situation than the one for which it was produced. Knuuttila and Morgan (2019) describe this as “deidealizing” the model and argue that this is no easy process of reversing the path of idealization in the context of model production. We generalize their approach beyond just models to “objects of scientific knowledge.” Just as they do not begin their consideration of deidealization as reversal of idealization, we do not begin our consideration of re-situation as processes related only to modeling in one context and application in a re-situated context. We expand beyond their “unit of analysis,” i.e., a source situation and a target situation. Finally, as noted above, we also shift the characterization of situation, and thus re-situation, from terms of “time, place and topic” to problem situations (Knuuttila & Morgan, 2019, p. 653).

Part of the philosophical and historiographic problem framed here, by means of a case study, is to ask whether re-situation can operate according to model-focused characterizations of idealization and deidealization if re-situations in empirical scientific practice tend to involve more extensive *bundles* of objects of knowledge. Our problem also concerns what difference the bundling of objects makes to the success of a re-situation, both how a bundle is structured as well as how its components are made to travel among various kinds of contexts in the co-production of scientific knowledge. We thus reframe the question posed in Howlett and Morgan (2011) of what makes scientific knowledge travel well in terms of an expanded characterization of objects of scientific knowledge and by refocusing attention on the social organization of workflows in scientific practice, from the more particular focus of the empirical (and policy) adequacy of model re-situation and application.

We cannot hope to “solve” the philosophical and historiographic problem at hand with a single case study, nor do we seek to generalize from one case. We instead aim to design a tactic of expanding units of analysis—extending Morgan’s (2014) characterizations of generic strategies—for studying re-situations that may apply to other cases and other situations. Our tactic involves extending the framing of the concept of re-situation from two to three sites (projects), expanding the objects of knowledge under consideration, and designing an enriched set of concepts regarding places and movements of knowledge through which to look at cases involving series or networks of projects. We are then enabled to suggest some empirical hypotheses about our case that might also apply in other cases, e.g., that skirmishes tend to escalate workflow complexity, not merely change them due to re-situation. Here, we aim to illustrate the tactic with a generative case study that we think can serve as a platform to make a start on characterizing re-situation and its objects of scientific knowledge in the hope that larger-scale comparative projects might attempt more general answers.

### Structure of the article

We structure our analysis in the following five sections. In section two, we contextualize the *originating project* (Rosenberg et al., 2002), focusing mainly on the workflow it originated. We offer the reader a detailed but necessary context on the computational methods (i.e., software renderings and the strengths and limitations of how software models genetic structure) which allowed the authors to see the presence of clusters in a dataset representing multiple populations across the world. In section three, we briefly outline and reflect on the workflow produced by the *challenging project* (Serre & Pääbo, 2004) to criticize the workflow used by Rosenberg et al. (2002) and their findings. In section four, we outline how the *response project* (Rosenberg et al., 2005) had to produce a larger workflow in order to explain that the discrepancies between the original and challenging projects were the result of the differences in the models, datasets and software configurations used to generate the different findings. Likewise, we address how the team proposed a resolution to the skirmish as clusters *and* clines rather than clusters *versus* clines. In the fifth section, we map out ways in which the skirmish can be considered to have reached an end despite continuations which signal that it is still being re-situated in meaningful ways. In the concluding section we provide the reader with final remarks about what this episode tells us about RSK more broadly.

## Seeing clusters in the originating project

As mentioned in the previous section, the most general finding in Rosenberg et al. (2002) was that the statistical partitioning of genomic data from 1056 individuals around the globe showed a pattern of similarity (clusters) reflecting continental divisions of our planet. The key to the finding was an approach to discovering clusters detectable by the software program STRUCTURE. Just what sorts of results the application of STRUCTURE to data of the kind gathered by Rosenberg et al. (2002) can produce became the subject of much discussion in the field (Lawson et al., 2018).

STRUCTURE uses Bayesian methods to assign individuals in a sample to source populations (Pritchard et al., 2000). Prior to this Bayesian approach, forerunners had developed related maximum likelihood approaches to population mixture and assignment (see Novembre, 2016). Because STRUCTURE’s methods are Bayesian, the authors of the most recent software documentation (version 2.3) caution that:

While the computational approaches implemented here are fairly powerful, some care is needed in running the program in order to ensure sensible answers. For example, it is not possible to determine suitable run-lengths theoretically, and this requires some *experimentation* on the part of the user. (Pritchard et al. 2010, p. 4, our emphasis)

Because there is an experimental art to running software of this kind (Markov Chain Monte Carlo simulation in a Bayesian analysis), re-situating the software in different project settings where researchers with different “artistry” or operating choices can create situations with different problems of interpretation of results, even when the exact same dataset is used as a starting resource. The same point holds with even more force for other scientists implementing the same algorithmic methods in software of their own design. Moreover, the complexity of the software (in terms of the number of parameters needing to be set in order to run it) means that it can be hard to compare findings from different analyses of even very similar datasets. Merely knowing that STRUCTURE was used to analyze a dataset is insufficient specification of the analysis performed. With a program setting, for example, the software allows for “admixture,” in which an individual’s genome is permitted to come from more than one source population, so individuals can be assigned to more than one population *proportional* to fractions of their genotypes assignable to different source populations. Findings do not always travel through communities together with specifications of the models, datasets, or software used to generate them, let alone particular parameter settings and software run choices.

The documentation for version 2.3 describes STRUCTURE as “a model-based clustering method for inferring population structure using genotype data …” (Pritchard et al., 2010, p. 3). To say that the method is “model-based” means in part that the software begins with an assumption or “model” of how many clusters, K, there are in a sample. An example of a non-model based method is Principal Components Analysis (PCA), which derives from a much older descriptive statistical technique for “dimensional reduction” to re-describe sets of data variables in terms of a “reduced” set of variables, or “principal components.” PCA came to rival and compete with model-based methods such as STRUCTURE’s cluster analysis in the research programs of human population genomic studies (HPG) of variation (HPGA, HPGB, HPGE, and HPGF).^21^ PCA constructs a new representation of the variation in a dataset in terms of a set of constructed variables, where the first one (first principal component) describes the most variation in the dataset as a linear combination of the original genotype variables. The second principal component describes the second-most amount of variation in the dataset as a dimension orthogonal to the first, and so on to a potentially infinite number of principal components (or until a vanishingly small proportion of variance is left to be accounted for). In short, PCA is a *descriptive* technique that can re-represent the variation present in any dataset with any number of variables. If the data—as described by any of the first few principal components—“clusters,” i.e., clumps in one part of that reduced dimension represented in a diagram plotting the data with principal components as axes, the PCA approach can be said to detect clusters. Just as there is an “art” to running STRUCTURE, there is an art to seeing “clusters” according to PCA and an art to interpreting PC variables—what principal component variables “mean”—in terms of the “loadings” of the original variables in the PC variables. Furthermore, PCA by itself does not identify clustering in the data, it allows human viewers of the plot to see clusters in the data or to use some further quantitative criterion or statistical test to detect clusters systematically. Since there is an “art” to criterion choice, the threat of apparent “circularity” applies to the use of descriptive, non-model procedures just as it does to model-based ones, although the displacement of the “art” or “interpretive judgment” step from the core algorithm or software onto the human viewer of a visual output or choice of statistical criterion of clusteredness supports a different rhetorical frame.^22^

Because the methods of STRUCTURE are Bayesian, a “run” of the software is really a complex, iterative process of provisionally assigning individuals to clusters using a set of assigned prior probabilities of cluster membership for each sample individual’s data. The software applies a population genetics model to calculate posterior probabilities, updating the prior probability values that result. Then the software feeds these updated values back through the algorithm again for some long number of iterations. The initial assignments might be “random.” The number of iterations used for Rosenberg et al. (2002) was 10,000 (see Supplement), following a “burn-in” length of 20,000, i.e., 20,000 iterative steps from the random starting assignments are thrown out and the next 10,000 are used as “the run.” The result is a set of “parameter” estimates that describe the distribution of cluster assignments of every individual (record) in the dataset.

The following passage in the software documentation captures quite well the protocol of exploration:

The program is started from a random configuration, and from there takes a series of steps through the parameter space, each of which depends (only) on the parameter values at the previous step. This procedure induces correlations between the state of the Markov chain at different points during the run. The hope is that by running the simulation for long enough, the correlations will be negligible.

There are two issues to worry about: (1) burnin length: how long to run the simulation before collecting data to minimize the effect of the starting configuration, and (2) how long to run the simulation after the burnin to get accurate parameter estimates. To choose an appropriate burnin length, it is really helpful to look at the values of summary statistics that are printed out by the program (eg α, *F*, the divergence distances among populations *D*_i,j_, and the likelihood) to see whether they appear to have converged. Typically a burnin of 10,000—100,000 is more than adequate.

To choose an appropriate run length, you will need to do several runs at each *K*, possibly of different lengths, and see whether you get consistent answers. Typically, you can get good estimates of the parameter values (*P* and *Q*) with runs of 10,000–100,000 steps, but accurate estimation of Pr(*X*|*K*) may require longer runs. In practice your run length may be determined by your computer speed and patience as much as anything else. If you are dealing with extremely large data sets and are frustrated with the run times, you might try trimming both the length of the runs, and the number of markers/individuals, at least for exploratory analyses. (Pritchard et al. 2010, p. 14)

A model of a whole population, such as the global human population represented by the HGDP-CEPH sample set, as having K = 2 clusters would assign individuals from a sample to 2 groups. A model with K = 3, would assign individuals to 3 groups. And so on. If the model also allows admixture, individuals could be assigned to as many as all K of the model’s “clusters” or as few as 1 cluster, in proportions reflecting similarities of portions of their genotyped fragments of genomes (the collection of autosomal microsatellites sequenced for the dataset) with other members of their cluster(s). Pritchard, Wen and Falush describe the basic model assumption underlying the approach STRUCTURE takes as follows:

Briefly, we assume a model in which there are *K* populations (where *K* may be unknown), each of which is characterized by a set of allele frequencies at each locus. Individuals in the sample are assigned (probabilistically) to populations, or jointly to two or more populations if their genotypes indicate that they are admixed. It is assumed that within populations, the loci are at Hardy-Weinberg equilibrium, and linkage equilibrium. Loosely speaking, individuals are assigned to populations in such a way as to achieve this. (Pritchard et al. 2010, p. 4)

The key here is that K may be unknown, yet the method is model-based, so a value for K must be assumed for the software to operate. What this means in practice is that the software is used in an *exploratory* way, by setting K to each of a series of values, 1, 2, 3, … K, in a series of computer “runs” and then interpreting the results of each run in terms of the larger context of an exploration of a series of clustering relationships.^23^ It’s a sort of “guess and check” method of exploration: guess that K might be some number, then check the results of assuming K is that value rather than some other value to see if the results make more sense under one model or another. Rosenberg et al. (2002) report results for K = 2 up to K = 6.^24^

Once assignment into clusters based on the genotype data has been made, *then* the researchers look at the named populations from which the samples were drawn. These population names were assigned to samples either by the sample collectors or by self-identification of the sample subjects or, in some cases, renamed by the researchers when configuring and analyzing the data, e.g., if they merged two samples to make one “population.”^25^ “Looking at” (visualizing) the results can be done partially by computer as well. Rosenberg, the lead author on the target article we are discussing, designed a software program, DISTRUCT, to display the results of STRUCTURE’s population assignments, applying the population labels to individuals grouped by clusters, where clusters as sources of (proportions of) genotypes are uniquely colored and each individual is arrayed in the bar chart according to that proportionality (see Rosenberg, 2004).

Many of the critiques and challenges to the approach to discovering clusters in Rosenberg et al. (2002), as mentioned earlier, appear rooted in the idea that the method is circular: it fixes the number of clusters and then “discovers” that humans can be clustered into that number of clusters. More insidiously still, the method finds that K = 5 is the best fit (on grounds of geographic distribution) and the particular 5 happen to be continental. Critics point out this roughly matches eighteenth and nineteenth century conceptions of the distribution of human “races” (see our arguments in Sect. 1 and Footnotes 20, 37). From what we described above of the method, it should be clear that that is *not* how it works because the method is used in an *exploratory* rather than *confirmatory* way. The complexity and sophistication of the *approach* using STRUCTURE as a *method*, the eye-candy appeal of the *visualizations* produced by DISTRUCT, the expertise required to understand the *models*, the opacity of the *sample* collection, and the intricacy of *dataset* assembly, all contribute to the difficulties of successfully re-situating any of these objects of knowledge for incorporation into new workflows, for challenging their production, and for interpreting *findings* within and outside academia. Moreover, many of the details needed for successful re-situation are hidden from view in supplements to papers, on websites, or in information only traceable by long chains of digital references back to online databases.

In February 2003, only a few months after publication of the Rosenberg et al. (2002) paper in a December issue of *Science*, Laurent Excoffier and Grant Hamilton of the Computational and Molecular Population Genetics Laboratory of the University of Bern, Switzerland, submitted a “Technical Comment” in *Science* regarding the 2002 paper (Excoffier & Hamilton, 2003).^26^ They focused on a seemingly small detail in the original study, that the amount of between-region differences (3–5%) was half that of previous studies, though still in line with the general pattern of findings since Lewontin (1972), of much smaller between-region than within-region differences. By reexamining the data of the originating project, alongside other studies and using a different “stepwise mutation model prevailing at STR loci,” Excoffier and Hamilton “reestimated components of genetic variance under the same hierarchical population structure used by Rosenberg et al.” The reanalysis also relied on a model of Excoffier’s from a 1996 paper. They got different results from Rosenberg et al. (2002)—results in agreement with the amount of between-regions variance in results prior to the Rosenberg et al. project, using different data and different approaches. They went on to argue that appropriate interpretation of the data depends on using an appropriate model for mutation at the loci analyzed. The upshot of the comment was that careful handling (with the use of appropriate models) of the Rosenberg et al. (2002) dataset is important “for estimating other important parameters of human population history.” (Excoffier & Hamilton, 2003, p. 1877b).

Rosenberg et al. (2003) reflected on this technical comment with a response comment of their own (p. 1877c). They pointed out that Excoffier and Hamilton analyzed only a subset of the Rosenberg et al. (2002) data (in order to render population samples more “uniform”) and treated them differently: as identified alleles that could evolve stepwise from, and back to, *particular* nucleotide sequences, e.g., from A to C and then back to A at a particular nucleotide site, rather than in terms of “indicators” of the presence or absence of a whole allele (microsatellite sequence). The fact that there were *multiple* differences (in terms of objects of knowledge deployed) between the two projects means that interpreting the differences in findings could not be directly attributable to a sole source of difference in their workflows, e.g., due to the change of mutation model from allele-wise to nucleotide-stepwise, or due instead to changes in the dataset. We think this technical challenge sets up the more substantial 2004 challenge in the skirmish due to this complexity of the re-situation of the dataset from original to challenge projects.

Rosenberg et al. (2003) go on to argue that the “stepwise mutation model” used by Excoffier and Hamilton “cannot be regarded as the “right mutation model”” (p. 1877c), particularly because their use of “indicator” names for alleles at loci does not admit of “stepwise” mutational change or back-mutation at nucleotide sites. The details of this technical exchange are not so important for our story of re-situation in the skirmish except to say that it highlights a feature of how challenges to the articulated workflows resulting in Rosenberg et al. (2002) would need to be dealt with: they represent disruptions that require workflow modifications.^27^ If you want to use a stepwise mutation model, you must code allele data by nucleotide sequence rather than with indicator names, but to do that, you must generate the raw nucleotide sequencing dataset corresponding to an indicator-coded dataset. This technical mini “skirmish” in 2003 signals a theme that emerged in the larger skirmish of interest as well: there were so many “moving parts” to the re-situation phenomenon that in order to challenge and respond to the original paper required an *escalation* of work, which thus presented significant disruption to the continued use of previous workflows. We see this in Rosenberg et al. (2005), which had to greatly expand the dataset compared to the 2002 study, use more complicated models, and deploy updated software, all to tease apart both questions and approaches that were not clearly distinguished in the ways the challengers had brought their challenges (see Sect. 4 below).

Also in 2003, advances in the population genetics model and analytical software (STRUCTURE) were made by an overlapping team. This team included two members of the original STRUCTURE paper (i.e., Pritchard et al., 2000), one of whom was also a co-author of the original Rosenberg et al. (2002) empirical paper (Pritchard), and one new member: Daniel Falush (see Falush et al., 2003). These advances added capabilities to the software to allow linkage between loci. Including linkage permits the software to handle more complicated situations of population “admixture” and to detect more subtle population subdivisions. Interpreting admixture turned out to be a critical point of controversy as the skirmish unfolded.

In 2004, a subset of the authors of the original 2002 paper, led by a postdoc who had joined the Feldman laboratory at Stanford University (Sohini Ramachandran), contributed a paper regarding the robustness of the kinds of findings produced by the methods of the original paper. The new paper considered microsatellites on the X-chromosome in contrast to the autosomal data of the original paper (Ramachandran et al., 2004). These technical projects, such as extending results to the X chromosome or to include linkage, advanced alongside the skirmish emerging out of the technical comment and response. They figure in later stages of the skirmish we are presenting.

In the remaining sections, we look in detail at the issues raised in the unfolding skirmish, how they were addressed in later papers, and how the skirmish more or less “ended” with the 2005 paper. We caution against a categorical answer to the question whether the skirmish ended in 2005 because these sorts of scientific exchanges occur in a published literature. They are therefore subject to re-opening, re-interpretation, and outright forgetting or silencing. Historical processes can keep open or re-open the past, so it is only in a rough, tentative sense that we say the skirmish “ended.”

## Seeing clines, not clusters in the challenge project

David Serre and Svante Pääbo of the Max Planck Institute for Evolutionary Anthropology in Leipzig, Germany challenged the findings and interpretation in Rosenberg et al. (2002) in a paper submitted to *Genome Research* in March 2004 and published in June 2004. They argued that features of study design (sampling pattern) and model (assumption of correlated alleles) led to Rosenberg et al. (2002)’s finding that individual sample subjects can be assigned to discontinuous, continental-regional “clusters” of humans based solely on genotype.

The worry was that if clustering results are sensitive to sampling design as well as model choice, then the clusters found may signal only apparent population structure imposed by the study design rather than discovered as a biological reality. The model choice—correlated or uncorrelated alleles—reflects a difference in perspective on the processes distributing humans from an origin in Africa and then migration “out-of-Africa.” The correlated alleles assumption (which determines how the software analyzes genetic data) is backed by an “out-of-Africa” model that supposes the alleles of different populations are more or less correlated because the ancestors of the different populations shared ancestry in Africa (Cann et al., 1987). The uncorrelated alleles model starts from an “out-of-Africa” origin, but supposes that migration of relatively small groups is coupled with genetic drift so that, by the time contemporary populations are sampled, alleles from the shared ancestors have become effectively uncorrelated. There are models within models, or models layered on the deployment of other models, in the papers of this skirmish. It becomes a challenge to track them through re-situation among projects since some are presented as background presuppositions or common knowledge in human population genomics that may not need to be debated among specialists with shared expertise, while others are made the focus of explicit attention in the design of a project workflow.

A different study design, Serre and Pääbo argued, with more homogenous global sampling (i.e., similar sample sizes per geographic area), together with a model of uncorrelated alleles revealed stable individual assignments into clusters for 4 or more clusters, but unstable assignments for fewer clusters. That is, as the number of clusters (hypothesized population groupings) changed, individual assignments also changed. Below 4 clusters, the instability of individual assignments to clusters for different values of K was interpreted to mean that individuals were mostly “admixed” and thus, Serre and Pääbo argued, are better interpreted in terms of geographic “gradients of allele frequencies” rather than in terms of “discrete clusters.” They produced this alternative design in part by sub-sampling from the dataset of Rosenberg et al. (2002) to create a more homogeneous distribution of sample data, though with reduced overall sample sizes.

Serre and Pääbo studied the proposed alternative sampling design by using datasets from studies by Lynne Jorde at the University of Utah (Jorde et al., 1997) and by subsampling the dataset used by Rosenberg et al. (2002), so as to produce samples with similar sample sizes (5–8) that were geographically as homogeneously distributed globally as possible, given the limits of the original dataset (e.g., there were no “North American” samples included in the original HGDP-CEPH dataset). So, they “re-situated” the dataset of the original study, modified it to fit a workflow designed to investigate homogeneous global sampling, and found *clines*, not clusters.

Serre and Pääbo associated their finding of clines, not clusters, as in line with earlier interpretations, based on classical genotyping, including by Luigi Cavalli-Sforza, founder of the Stanford “school” of human population genetic studies (which includes several of the authors of the Rosenberg et al., 2002 project).^28^ Serre and Pääbo actually cite various works by Cavalli-Sforza’s group supporting both sides of the clines versus clusters interpretation of global human genetic diversity studies.

On the basis of their findings, Serre and Pääbo argued there is no reason to conclude that “major genetic discontinuities” exist between continents or “races,” in contradiction to the claim made by others that “the greatest genetic structure that exists in the human population occurs at the racial level” (Risch et al., 2002; cited in Serre & Pääbo, 2004: p. 1683). They do acknowledge, however, that geographic discontinuities might exist on more local scales and they might also exist, even if very small genetically, on a continental scale. They conclude: “on a worldwide scale, clines are a better representation of the human diversity than clades,^29^ and that continents do not represent more substantial discontinuities in such clines than many other geographical and cultural barriers.” (Serre & Pääbo, 2004, p. 1683).

## No, seeing (mostly) clusters in the response project

A year later, three of the authors of the original 7-author 2002 paper, again led by^30^ Rosenberg (then at the University of Michigan) published a paper in *PLoS Genetics* (Rosenberg et al., 2005) responding to the challenge posed by Serre and Pääbo (2004) in *Genome Research*. The new team included original members Feldman and Pritchard, plus new team members/co-authors: a computer scientist, Mahajan (at USC), Ramachandran (then still a postdoc at Stanford University), and Zhao (at Marshfield Medical Research Foundation). Weber, Cann, Kidd, and Zhivotovsky did not co-author the response paper.

Because Serre and Pääbo had criticized the sampling design (population-based rather than uniform-geographic) and the model (correlated rather than uncorrelated alleles) of the 2002 paper, their alternative 2004 workflow and analysis involved altering several objects of knowledge: the *datasets* used and the *model* deployed, while holding fixed the *software* (STRUCTURE), in order to see whether the original *findings* (continental clusters) are corroborated in the re-situated analysis.

This alternative analysis presented to the Rosenberg team a problematic re-situation. The assembly of several imported objects of knowledge into the new project situation of Serre and Pääbo for assessing the original published findings in the new context was problematic because the contributions of each object in the challenge project could not be studied separately. The individual effects of study design and model changes in the challenge paper on the findings could not be independently assessed in order to respond to the challenge. This kind of entanglement of changing configurations of objects of knowledge figured in the technical comment and response of 2003, as Rosenberg et al. (2005) noted:

Thus, although a difference in results was seen between the analyses in [Serre & Pääbo, 2004] and those in [Rosenberg et al., 2002], the attribution of this difference specifically to a difference in geographic dispersion or to a difference in assumptions about allele frequency correlations is problematic, because both of these variables differed between studies, as did the number of individuals. (Rosenberg et al., 2005, p. 0661)

Rosenberg et al. (2005) expanded their 2002 dataset in order to have enough markers (993 instead of 377) to evaluate these and other variables one by one: “sample size, number of loci, number of clusters, assumptions about correlations in allele frequencies across populations, and the geographic dispersion of the sample” (Rosenberg et al., 2005, p. 0660). They used linear regressions of each study design variable on a statistic they called “clusteredness” to evaluate the effect of each study design variable on its own, independently of the contributions of other variables in play in the re-situations of the dataset and model from 2002 to 2004 to 2005. They found that geographic dispersion (how samples are distributed geographically) had little effect on the degree of clustering discovered in the data. Thus, they corroborated the original findings, though the original dataset had again been re-situated and modified in the context of the response project.

More importantly, to achieve the sense in which this response project and paper (more or less) ended the skirmish, the authors argue that clines and clusters are *compatible perspectives* on the genetic structure of human population distributions geographically. In the paper’s synopsis, the authors claim:

Previously, it has been observed that when individual genomes are clustered solely by genetic similarity, individuals sort into broad clusters that correspond to large geographic regions. It has also been seen that allele frequencies tend to vary continuously across geographic space. These two perspectives seem to be contradictory, but in this article the authors show that they are indeed compatible. (Rosenberg et al., 2005, p. 0661)

The response paper renders choice between the contrasting interpretations moot by arguing for compatibility of perspectives at the same time it raises the bar considerably for how to evaluate conflict between the interpretations. While Serre and Pääbo (2004) had argued that the interpretations are incompatible, given the question, they suggested that there were really two questions on the table and Rosenberg et al. (2002) had addressed one of them while Serre and Pääbo had addressed the other. The point of skirmishing was, presumably, that there is great potential for generating study designs inappropriate to the intended question under study and therefore for misinterpretation of findings.

In expanding the scope of the original 2002 project in order to evaluate the individual contributions of study design variables to the findings, Rosenberg et al. (2005) rendered the apparently competing interpretations compatible and in the process substantially complicated the workflows of anyone who henceforth wants to contribute to research in this area. Participants in the specialty, going forward, would either have to (a) make assumptions in relation to interpretation much more explicit, in order to link a precisely stated question specifically to study design choices on each of the variables analyzed in Rosenberg et al. (2005), or (b) design studies large and comprehensive enough (as in the response paper itself) to evaluate the contributions of such variables in the way that the response project had done in order to support answers to a variety of questions. The first strategy would limit interpretation to a single, precisely characterized question so that general interpretations of the findings would not yield the conflict. To pursue the second strategy, participants would have to follow the path of the response paper itself, tracking precise changes in each object of knowledge resituated into new projects, in which the conflict resolves into compatibility but at the cost of a greatly expanded study design.^31^

An alternative reading of the situation in 2005, however, is also contained in the abstract of the response project’s paper. There, the authors point to a different framing of the question(s) under inquiry, expanding the nature of the original question to a more encompassing one regarding the *relationship* between genetics and geography:

Examination of the relationship between genetic and geographic distance supports a view in which the clusters arise not as an artifact of the sampling scheme, but from small discontinuous jumps in genetic distance for most population pairs on opposite sides of geographic barriers, in comparison with genetic distance for pairs on the same side. (Rosenberg et al., 2005, p. 0660)

At the very least, this framing raises the possibility of resolving the skirmish, not by choice of study design, or model, or workflow, but rather by renegotiating the formulation of the question as broad enough to encompass both questions and perspectives, so that the original and challenge designs are both embraced within the workflow of a “geogenetics” or a “genetic geography” and therefore rendering compatible the interpretations of seemingly irreconcilable findings.

Moreover, there is a third *way* in which the skirmish might reach a form of closure (for those directly involved in it) besides the above two readings that the skirmish “ended” in 2005, one that affords very different prospects for tracing the re-situation of findings and interpretations to challengers working *outside* the research specialty. This is a resolution that distinguishes human *population genomicists* from other interpreters of human geography, ancestry and race, i.e., challengers who are unprepared or uninterested to take on the specialist genomic work required to offer technical examinations in contexts of re-situated interpretations of findings. Serre and Pääbo had introduced a new way of viewing the kinds of projects that could be performed using the kinds of objects of knowledge and organized workflows of the Rosenberg et al. (2002) project. By sampling *populations*, genomic studies deliver *population structure* as the form of the findings. Their challenge project suggested that if you sample differently (without regard for “culturally defined” populations), you don’t discover these “theoretical” populations (clusters) from the genetic data, you find clines or geographic gradients of genetic variation.^32^ In other words, the challenge might continue to have “legs” despite the escalation in the 2005 response paper. This is because the response does *not* foreclose alternative interpretations of study design contrasts for interpreters who are not going to take advantage of those *particular* contrasts in the performance of *genomic* specialist technical work. This may lead to disparate interpretations of findings by *those who choose not to address the nuances* of the “population genomicist” response paper and the technical reconciliation of approaches and questions it offered. Differently put, such critics and challengers were not performing workflows that would be disrupted by the population genomicists’ responses to the challenge project inside the skirmish, so the challenge is not foreclosed to other modes and contexts of critique. So, the skirmish response via escalation of workflow cost and complexity has no bite beyond the reach of the need to perform such workflows in the first place. Thus, specialists with shared expertise in producing findings of these kinds can lose control of narratives constructed by means of those workflows when their findings travel beyond the bounds of their technical practice. If the scientific work of Rosenberg et al. (2002) and the challenge work of Serre and Pääbo (2004) disrupted or became relevant for the work of others, it was likely only in the sense of affording a new focus of ongoing critiques of interpretations of genetic work as “racist” or affording new fuel for their interpretations of genetic work as supporting biological race realism.

In the opening paragraph of their discussion section, Serre and Pääbo (2004, p. 1682) write:

[…] the discrete clusters described by Rosenberg et al. (2002) from analyzing more than one thousand individuals of the CEPH diversity panel *might be caused by discontinuities in the sampling*, because when samples that have equal numbers of individuals of each population are analyzed (Fig. 2), the inferred populations yielded by Structure do not match continents or geographical regions but represent theoretical “populations” in which all individuals show admixture to at least two such “populations.” (Serre & Pääbo, 2004, p. 1682, our emphasis)

This view of the interpretation of findings amounts to a kind of circularity charge, as we mentioned above: populations *are built into* the study design, so the findings are a kind of artifact of method, not a discovery of population structure in nature. Such a view resonates with many of the cultural critiques of an alleged association (as read by some audiences) of a concept of “race” with continent-scale differences among humans that emerged after the Rosenberg et al. (2002) study.

The circularity charge might be taken up in contexts where the sentiment expressed in the quotation above aligns with concerns about the characterization of human groups as biological populations, e.g. in some contexts outside the community of producers of such knowledge. In their article, Serre and Pääbo moved (perhaps inadvertently) the framework of “ancestry” into one of “race” and “ancestry” via an apparent *contrast* between competing interpretations of data in terms of ancestry gradients and discrete racial populations. This may have contributed to the sense that work such as Rosenberg et al. (2002) is inherently aligned with a racialist interpretation while gradient work can align with a non- or anti-racialist interpretation. In any case, the challenge project seems to have opened possibilities for linking both critiques and endorsements of the relevance or reality of biological race to the kind of work represented by Rosenberg et al. (2002). The opening created a fresh opportunity for controversy about how to contextualize their findings in academic and non-academic networks of reflection around race.

That is, there are on-going questions about the *further* re-situation of objects of knowledge arising from this kind of study beyond the context of this skirmish and, indeed, beyond all of the scientific specialties that might find interest in re-situating the methods and workflows of Rosenberg et al. (2002). These may be contexts in which the *findings* are *blocked* (or attempted to be blocked) from re-situation into settings where concepts of race and other modes of essentializing human variation as group differences have specific (positive or negative) value and impact, such as forensics, biomedicine, economics, politics, and questions of social order.

This third way of assessing yet not closing the skirmish was *not* the view expressed by Serre and Pääbo, who, a paragraph later in their discussion of their alternative findings and interpretation, suggested that both approaches and interpretations are “valid” because they are designed to answer different questions:

Thus, whereas Rosenberg’s group investigates whether individuals can be assigned to culturally predefined populations on the basis of their genotypes, we investigate the patterns of relatedness across the human gene pool. The goals of the two approaches are both valid but clearly distinct. (Serre & Pääbo, 2004, p. 1683)

This duality of questions leaves open the possibility of assessing the skirmish yet leaving open the possible continuing critique of population genomics. They go on to draw a lesson for attempted applications (i.e., re-situations) of such findings and interpretations from their methodological intervention to other contexts of use, such as biomedicine and forensics:

[…] it is important to stress that when the goal of a study is to identify the geographical origin of one individual (e.g., in forensics) by his/her genotype, the results will be very dependent on the populations used as references and to their genetic relatedness with the sample investigated. (Serre & Pääbo, 2004, p. 1683)

This is a conclusion with which we presume Rosenberg et al. (2002) and (2005) would not disagree even if it wasn’t stated in either of the papers. However, paths leading out from the findings and interpretations to this (presumably) shared lesson, are quite different, and it might be the *pathways of reasoning* rather than specific objects of knowledge that are re-situated into popular media and critique. For Serre and Pääbo, the path to the lesson stems from a potential failure to align study design with questions asked. For Rosenberg’s team, the path stems from taking care in organizing workflows to answer the questions asked. The 2002 paper asked and answered one question: can continent-scale human population structure be detected in the HGDP-CEPH samples using genotype data alone? To answer different, additional questions, they expanded the 2002 dataset to assess study design *trade-offs* along with expanded model and software capacity and functionality to answer multiple questions, rather than simply designing a different workflow to answer a different question, as Serre and Pääbo had done.

## When did the skirmish end?

Did the skirmish end with Rosenberg et al. (2005)? Our narrative so far suggests that the escalation of effort and complexity of work in Rosenberg et al. (2005) that was needed to evaluate the challenge of Serre and Pääbo (2004), together with the argument for compatibility of seeing clusters and clines at the same time, in the same data, suggest that it did “end” with them. Serre, for example, cited his 2004 paper in papers he co-authored later on (Serre & Hudson, 2006; Serre et al., 2008), but neither paper presented the 2004 paper as a challenge to Rosenberg et al. (2002). Serre and Hudson (2006, p. 446), for example, merely state the alternative 2004 finding that: “… the geographic distribution of the diversity seems to best be explained by large gradients of allele frequency rather than by well-defined and separated clades corresponding to continental or “racial” entities,” citing the 2004 paper and then adding a “but see also” with a citation to Rosenberg et al. (2005). The remark is prefaced by the opening of the paragraph with an acknowledgment of the “classical” (i.e., 1960s-1990s) finding and interpretation offered in Rosenberg et al. (2002): “Analyses of the genetic diversity among humans reveal very little differentiation among populations. Grouping individuals according to their geographical origin is feasible but requires large data sets of highly informative genetic markers (see, e.g., 62 [Rosenberg et al., 2002]).” Pääbo appears not to have cited the 2004 paper since its publication.^33^

More recently, Peter et al. (2020) give a historical sketch, suggesting that the skirmish had a long tail or shadow after 2005, which can be read as a pathway toward resolution. Their opening paragraphs reconstruct the history of studies of “human genetic diversity” in three phases: from classical blood group and allozyme loci (citing Barbujani & Sokal, 1990 and Cavalli-Sforza et al., 1994), to microsatellite marker panels (citing Rosenberg et al., 2002), to the skirmish we describe about clusters versus clines (citing Serre & Pääbo, 2004; Rosenberg et al., 2005; and also citing two papers we haven’t discussed here by Frantz et al. (2009), Perez et al. (2018), projects that contribute to the specialty but don’t address the skirmish).

Whether the Peter et al. (2020) narrative implies the skirmish lingered on after 2005 and/or rather that it helped prompt a new and different research program with a different focus is difficult to confirm with the evidence we have collected on this case study so far. This may be better clarified by interviews of participants (in progress). What we want to signal here, in this section, is that a new(ish) kind of research program was pursued by several groups aiming to more explicitly bring genetic and geographic dimensions together, in a nuanced way beyond simply referring to the population labels on samples so as to “interpret” the *geography* of human genetic diversity.

This new research program, requiring new objects of knowledge and new workflows, emerged and developed over the decade or so following the papers from 2002 to 2005 that we considered as the “core” of the skirmish. Although themes of human genetics and geography go back, really, to nearly the beginnings of genetics in the twentieth century (e.g., see Gannett & Griesemer, 2004 for discussion of early work on human blood group distribution studies), the point is that new methods in both genetics and studies of human geography, demography, and other related disciplines transformed research programs investigating the relationship in ways that make it challenging to disentangle the skirmish from its larger context. The new methods and approaches of some papers cited and discussed by Peter et al. (2020) attempted to “incorporate geography directly” into the “analytical methods to represent population structure.” The authors’ goal is to deploy such methods to “visualize how human genetic diversity is geographically structured” (p. 943).

Indeed, geography and spatial distribution are key directions approached in some of the research reviewed and cited by Peter et al. (2020), suggesting that life-scientists took aim at (or at least had the side-effect of) resolving the skirmish by moving beyond it to make the *linkage* between genetics and geography the explicit focus of research. For example, Ramachandran et al. (2005) emphasized the relationship of genetic and geographic distance. Novembre et al. (2008) argued that genes “mirror geography.” Frantz et al. (2009) tested the robustness of correlating genetic divisions with “landscape features” (beyond humans). Novembre and Peter (2016) reviewed advances on the identification of “fine-scale” human population structure. Bradburd et al. (2018) assessed how discontinuous sampling and geographic “isolation by distance” impact inference and visualization of population structure (beyond humans). Perez et al. (2018) also argued that analytical tools (software) can be extremely impacted by isolation by distance. In these papers, “isolation by distance” is a concept articulated in a population genetic model, tracing back to original work by Sewall Wright that frames the way population geneticists understand, interpret, and investigate clinal patterns of variation (Wright, 1943). Consideration of this model in juxtaposition to clusters or “structure” signals a continuation of the theme of the skirmish but, in most of these cases, by other or newly developed means, including new data (and approaches to data collection), new ways of visualizing space and geography (or in some cases geographic dimensions), new models, and new software. Perhaps it makes sense to say that when most or all of the objects of knowledge in a workflow must be replaced in order to continue a research program disrupted by a skirmish, the skirmish is effectively “over.” Differently put, in asking what may seem like a closely related question, if a research program must replace most of the objects of knowledge it uses to answer such questions, we can recognize various versions of what might as well count as “ends” of the skirmish, even if it is not “the” end.

An interesting example of how a skirmish that ended can be revived is the case of how David Reich picked up and reframed our 2002–2005 skirmish in his recent book on ancient DNA (Reich, 2018a). Rather than using this skirmish to discuss further modeling and analytical dimensions vis-à-vis his own, more recent contributions to HPGA studies, Reich emphasized the re-situation of findings in Rosenberg et al. (2002) to illustrate a *different* skirmish in which Reich himself became involved after about 2007. For Reich, “Feldman's study” (i.e., Rosenberg et al., 2002) and the earlier skirmish represented a “first major engagement” between the genomic revolution and an alleged “anthropological orthodoxy.” As discussed above, Serre and Pääbo’s challenge project raised interest in the character and interpretation of evidence about the structure of human populations. Reich notes that Serre and Pääbo questioned the (population-based) “nonrandom” sampling of Rosenberg et al. (2002) (see Reich, 2018a, pp. 251–252). Reich *also* points to Rosenberg et al. (2005) as responding to Serre and Pääbo’s challenge by showing that while population-based nonrandom sampling does not account for “most” of the variation, “substantial clustering is observed […] even when,” as Serre and Pääbo had attempted, “repeating analysis on geographically more evenly distributed sets of samples” (Reich, 2018a, p. 252). In other words, Rosenberg et al. (2005) closed the skirmish on clusters versus clines, but Reich used it to open a wider skirmish about HPGA versus what he called “anthropological orthodoxy.”

Although we cannot here pursue this re-situation of the whole earlier skirmish into the context of genetic versus biological anthropology ancestry studies, we think that characterizing the earlier skirmish as representative of an anthropological orthodoxy is misleading. After all, Pääbo was trained as an MD and biologist with post-doctoral training in Allan C. Wilson’s laboratory,^34^ and his research work at the time—as researcher and director of the Max Planck Institute for Evolutionary Anthropology—was highly interdisciplinary in scope and aims: hardly a likely source of anthropological *orthodoxy*. The uptake or pull of our skirmish into the context of ancient DNA studies in the later 2000s illustrates how one skirmish can end up enrolled into a different or wider one about correlations between race ideas and human population genomic origins. Needless to say, this is a high-stakes debate for Reich (see Reich, 2018a, in particular Chapter 11; Kahn et al., 2018; Reich, 2018b). For RSK purposes, it suggests a way in which even more inclusive “objects of knowledge,” such as whole episodes or sequences of published work, like this skirmish, may be subjected to re-situation.

## Conclusion

The point of initiating the skirmish was to challenge the further circulation of particular objects of knowledge within the specialist community and perhaps block certain conclusions and interpretations beyond it, such as the race-related interpretations that did appear in the popular media. The skirmish, in other words, contested the original workflow because of the objects it re-situated and the findings their deployment produced. The challenge was not only to the question of whether the findings should circulate, and potentially travel as facts, but whether other objects of knowledge—the model(s), the dataset and the software (as then configured)—should circulate as well, as traveling “companions” (see Howlett & Morgan, 2011). The skirmish challenge therefore questioned the value of circulating and further using many of the objects of knowledge originally presented. Moreover, the challenge represented a disruption to the original workflow as some members of the original team had to consider whether and how to respond to the challenge, rather than simply carrying on with what they had been doing.

The skirmish ended in the sense that while work on the various projects that became engaged in the skirmish continued, they ceased to carry on the disputes in that form, and forum and citations to the collection of papers “in” the skirmish shifted to other topics, projects, and research programs. To the extent that the study and workflow designs of the original, challenge, or response projects became models for work in the field, the close of the skirmish figures in the justification of continuing work on either the original/response or challenge lines. Additionally, misunderstandings, misrepresentations, or alternative interpretations of the skirmish have led to other kinds of critical work beyond the limits of the technical specialists. In any of these ways, new work takes the skirmish itself as settled, though who “won” is open to interpretation.

We cannot answer here the question whether the challenge paper became viewed as *closing down* a research program on grounds of circularity of method or the response paper became viewed as categorically ending the skirmish through escalation. We also cannot yet settle the question whether the episode was merely a *technical iteration* in a larger quest to develop *the methods* of HPGA studies in the face of rapidly advancing understanding of the objects of knowledge—the models, the data, the software—in the specialty. It is striking that the approach to HPGA pioneered in Rosenberg et al. (2002) took place alongside technological advances in DNA sampling and sequencing and advances in other disciplines bearing on the history of the peopling of the earth (e.g., anthropology, archaeology, linguistics, and paleontology, to name a few). The reason we cannot answer is that it seems likely both answers are correct.

Within HPGA studies, the literature after 2005 blossomed into a tangled web of data-centric studies, model developments in several directions, software proliferation, rivalry among competing analytical approaches, and several game-changing technological developments (inexpensive whole-genome sequencing and successful extraction of ancient DNA suitable for SNP analysis from human fossil remains, to name two). Outside HPGA studies, in popular media and some academic social science criticism, the critiques of STRUCTURE and genetic clusters as a way of understanding human groups became linked with a rising critique of racism that took on a life of its own (see Sect. 1 and Footnote 20), in that these critical contexts of re-situation of findings advanced without continued detailed analysis of the other objects of knowledge that usually traveled with the findings inside HPGA studies.^35^

We suggest the potential for misunderstanding and misrepresentation *on the part of practitioners of HPGA studies* and other audiences demanded a response by Rosenberg et al*.* to clarify how their original workflows (including sampling and study design) led to their published findings and interpretation. The challenge disrupted their workflow by re-situating their data, models and findings in alternative workflows, leading in turn to alternative findings and interpretations. Their response was not designed to address critiques that did not depend on the same sorts of workflows and re-situations performed by Serre and Pääbo, and indeed, may have had the effect of silencing the other objects of knowledge so that the findings travelled *outside the specialty* with misleading or simplified claims about “study design,” receiving interpretations in terms of “race” rather than the producers’ preferred technical understanding of “ancestry” (Wade, 2002, 2014; Coop et al., 2014; Feldman, 2014; see also Wills, 2017). Importantly, the challenge paper in the skirmish pointed to a further potential consequence for RSK beyond the community of specialists: the circularity charge, arising from a characterization of the study design, which seemed to escalate debates over interpretation to the centuries-old understanding of race, so that the skirmish revealed what was already at stake in a much larger war.

Returning to our opening question about RSK, we asked whether re-situation can operate according to a model-centered characterization of idealization and deidealization between contexts of production and application or use. We showed in our case study that re-situations in a skirmish about HGPA studies, from an original to challenge to response projects involved more extensive bundles of objects of knowledge of a wide variety of kinds: models, datasets, analytical and visualization software, and findings. The case study demonstrated that a precise, accurate understanding of re-situation involves the whole bundle because: (a) more than one object of knowledge was involved in each step of the “stepping stones” (see Morgan, 2014 and Footnote 3), and that these objects *interacted* in important ways during the re-situations, and (b) in considering more than one step between two stepping-stones, it became evident that different parts of the bundle of objects were saliently re-situated and that additional stepping stones (e.g., from other datasets, other models) became entangled in the skirmish, so as to reveal larger stakes, larger programs, and more complicated trade-offs.

We conclude that it is worthwhile to expand the unit of analysis from single-steps between two stones (local projects) to at least the two-step, three-stone skirmish described in our case study. We also envision expanding the analysis of idealization/deidealization, travel, abstraction/concretion of single objects of knowledge, such as a dataset or a model or a finding, to bundles of various kinds of objects of scientific knowledge. These two enrichments of the emerging literature on RSK not only give a richer picture of what these phenomena are like, but also afford a potentially more precise and accurate account of historical and contemporary cases of the dynamics of research work in scientific practices beyond HPGA.

## Data Availability

This study did not involve the generation of new data.

## Footnotes

1 The language of “push and pull” to describe re-situation is due to historian Jason Oakes.

2 We take facts to be findings accepted by a community, so a finding may only be a candidate fact. A fact can also be “demoted” or returned to the status of finding when challenges to its “facticity” are made. See Griesemer (2020) for further discussion. A separate paper on re-situation of scientific knowledge will discuss objects of knowledge in philosophical and historiographic perspectives.

3 Morgan (2014) uses the metaphors of stepping stones and bridges to describe the “local to local” movements of knowledge from one setting to another, in contrast to the generalization of knowledge by abstraction from local situations and applications elsewhere, using a metaphor of ladders (see also Cartwright, 2012).

4 We are aware that a likely significant driver of re-situation is the role of norms and values, both constitutive and contextual (Longino 1990). Other drivers, of course, are workflows, repertoires, institutions, and common knowledge in scientific work. On repertoires, see Ankeny and Leonelli (2016). Our insights owe a large debt to Elihu M. Gerson’s work, e.g., see his (2013, 2014) on institutions, common knowledge and meta-work in the integration of specialties; (2008) on coordination mechanisms that facilitate cooperative work; for re-situation of data collection technologies; and work in progress on institutions and the situated repertoires enacted in performances of scientific work.

5 Re-situation for us means re-siting a problem structure in virtue of re-situating one or more objects of knowledge from the setting of one workflow or research problem to another. Knuuttila and Morgan (2019, p. 653) characterize “situation” in terms of “time, place and topic.” We consider “time, place and topic” a minimal characterization of a “setting,” where a setting is a context in which work is (to be) done. A richer characterization of settings would include the particular organizations and institutions involved in specifying local “places” at times and on topics. We reserve “situation” for *a specific problem in a setting*, where a problem can be a theoretical or conceptual one about the topic of research, but could just as likely be a methodological one. Methodological problems include disruptions of local workflows in operation (or being designed or under construction) that had been successful in getting scientific work done in a local setting. Disruption (or “improvements”) to workflows are changes of “method” needed or desired in order to address research questions on a topic. Knuuttila and Morgan (2019, p. 656) offer some examples that *are* in line with our specification of “situation.”

6 For biomedical applications we will refer to HPGB studies; for forensics we will refer to HPGF studies; for applications in evolutionary biology beyond just humans, including studies of adaptation and natural selection, we will refer to HPGE studies. Thanks to Rasmus Winther for helpful discussion of these distinctions.

7 Indeed, some of the changing names given to the topic of study mark re-situations as well, e.g., when new methods for studying ancient DNA led to blending datasets built from ancient and contemporary samples to infer global or regional human migrations (e.g., Alan C. Wilson’s laboratory in the 1980s, David G. Smith’s laboratory in the 1990s, or David Reich’s laboratory in the 20-teens; see Cann et al. (1987), Kaestle and Smith (2001), Reich et al. (2012) respectively). Another re-situation (of methods) came with the application of phylogenetic methods to reconstructing human ancestry in the 1960s (e.g., Cavalli-Sforza & Edwards, 1967; Edwards & Cavalli-Sforza, 1964). See Winther (2018) for papers and commentary on A. W. F. Edwards’ work. The re-conceptualization of the field of population genetics as population genomics in the 2000s also marks a broad re-situation (of population genetic models into molecular genomic practices) following the genome sequencing revolution from the 1990s (Charlesworth, 2010); Donoghue & Love, in press).

8 We study *human* population genomics studies because, when the stakes are high due to application to humans, every aspect of the studies is subject to heightened scrutiny and every object of knowledge is subject to re-situation. We study human population genomic *ancestry* studies (HPGA) because it is a smaller mountain to climb than human population genomic *biomedical* studies (HPGB). For a detailed historical study of the “foothills” of HPGB studies leading from the population *genetics* of a single mutation in the *CCR5* gene (of interest in the study of immunology and HIV/AIDS) to the transition to population *genomics*, see Jackson (2015). For a study of micro-re-situation of objects of knowledge in human population genomic *forensic* studies (HPGF), see M’charek (2005). Once our analytical tools for the former are in place, we will attempt to scale the higher peaks in the RSK (Griesemer & Barragán, 2018).

9 We toyed with various terms to name these “engagements”: conflict, controversy, counterpoint, debate, engagement, episode, exchange, scuffle, or skirmish. Although we don’t like war metaphors, we adopt the term ‘skirmish’ because it also conveys a sense of disagreement, dispute or conflict that could be part of a wider conflict, engagement, “battle,” or “war,” though without any commitment to how major or minor the skirmish was or whether one “side” or another should be regarded as having won or lost. We seek to characterize exchanges in the published literature that are relatively contained in temporal duration and number of participating parties and to use them to understand re-situation phenomena.

10 We treat research projects (potentially) as parts of research programs, so that the latter can be distributed among groups, teams, or places over multiple projects. A project is something done “locally,” either in a single locality (like a laboratory) or by a single team, even if the team members are distributed in different localities. This means we also distinguish place from space, and in this perspective,’geography’ can apply to either place or space/location.

11 The data involved autosomal microsatellite loci. Autosomes are the 22 pairs of non-sex chromosomes in humans. Microsatellites are short tandem repeats of nucleotides or STRs, which a wide body of evidence suggests are mainly “neutral” sites, i.e., not coding genes under natural selection. Microsatellite data was commonly used to study population variation, evolution, and ancestry before whole-genome sequencing became available around 2007. Microsatellite data was usually designed to serve as a random sample of loci across the whole genome.

12 A sixth cluster corresponded to a single, distinctive regional group within one continental region: the linguistically unique Kalash people of present-day Pakistan (see also Footnote 24).

13 See Griesemer (2020, Figure 1), for a generic representation of this kind of scientific workflow. The CEPH website describes the sample set origins as follows: “The goal of CEPH was to initiate and lead an international collaboration in order to establish the first genetic map of the human genome. To achieve this, a collection of DNAs from 40 large CEPH reference families has been made available to the scientific community. More than 120 collaborators were involved in the genotyping effort. The families’ genotypic data have been gathered at CEPH and made available in a central database. This led to the building of more than ten genetic linkage maps of the whole human genome in the 1990s.” The website goes on to describe the origin of the HGDP this way: “In the early 2000s, Professor Howard Cann collaborated with Professor Cavalli-Sforza from Stanford University (USA) to create the Human Genetic Diversity Panel-CEPH (HGDP-CEPH). The panel includes 1063 DNAs from 52 populations from all continents. Genetic material from the panel is distributed to researchers all over the world to be used in projects on different fields of population genetics.” Retrieved February 23, 2021, from http://www.cephb.fr/en/presentation_historique.php.

14 The genotype data derived from the cell line samples were supplied by the research arm of Marshfield Clinic, the home organization of one of the study’s co-authors: James Weber. Another co-author, Howard Cann, was located at CEPH (Rosenberg et al., 2002). For more on the journey traveled by this dataset, see Griesemer (2020).

15 We will report elsewhere on the HGDP sample subjects, where they live (or lived), and the samples and data from them that researchers collected or used. On HPGA datasets, see Griesemer (2020).

16 Elsewhere, we will discuss visualizations of data and findings as a kind of object of knowledge. Of particular interest in HPGA studies are maps of various kinds bringing genetics and geography data, models, and findings together to interpret human migration history and patterns.

17 Google Scholar search on March 9, 2021.

18 The likelihood is high of producers’ work being misappropriated so that they lose control of their narratives when their objects of knowledge are re-situated to popular media contexts. Wills (2017) explores the misappropriation of the rhetoric of “ancestry” as a reflection of “race” and the way in which such scientific narratives pass into popular media. DiMarco (2020) explores the history surrounding the publication of Cann et al. (1987) and the co-production of “mitochondrial Eve” by the joint work of scientists and popular media.

19 While Lewontin (1972) argued for similarity across “clusters” in the sense that most variation among humans is to be found in individual differences within populations and that much less variation distinguishes clusters, Rosenberg et al. (2002) pointed out that clusters can be distinguished, based on the small percentage of genetic variation between them, i.e., that the small variation between clusters is “real” and relevant for evaluating ancestry relations, even if not “significant” for interpreting human variation in racial terms.

20 Beyond the early references cited above, later readings and critiques of the findings in the article include, among others: Spencer, 2012; Templeton, 2013; Kaplan & Winther, 2013; Fujimura et al., 2014; Winther & Kaplan, 2013; Spencer, 2014; Winther, 2014; Edge & Rosenberg, 2015; Graves, 2015; Spencer, 2015; Glasgow et al., 2019.

21 Principal Components Analysis was so-named by statistician Harold Hotelling (1933). How PCA entered HPG is an interesting re-situation in itself but its analysis goes beyond the scope of this article.

22 Consideration of model-based vs non-model-based approaches to genetic structure of populations and reconstruction of human population genomic ancestry is another, wider debate than the case study skirmish we detail here.

23 In practice, it is still more complicated than this. For each K, a series of runs, say 10, might be produced and the most “informative” (according to a statistical criterion) or an average of the runs will be used to evaluate the fit of the data to that model value of K.

24 Jonathan Marks (2010, pp. 265–276) notes that at K = 2, the results roughly divide into Europe + Africa + Western Asia on the one hand and Far East + Oceania + New World on the other hand. At K = 5, the clusters loosely correspond to continental areas, but at K = 6, the 6th additional cluster is the Kalash people of Pakistan (see also Footnote 12 above). Marks points out that it is not “racially commonsensical” to draw conclusions about race from such cluster analysis because that would imply the Kalash have equal continental “standing” as a race to “African” or “Asian.” We agree. Note, though, that Rosenberg et al. (2002) themselves do not draw racial conclusions on this point. They point only to the possibility that the genetic distinctness of the Kalash may make their linguistic isolation from neighboring groups a cultural signifier of equal consequence for genetic ancestry as continental geography seems to be at K = 5.

25 Population names reflect geographical or political regions either self-designated or by sample collectors and can thus reflect a range of values and choices that frame contexts of population-identification. See Griesemer (2020) for examples of reassignment of samples to populations and the reasoning behind it. The dataset used by Rosenberg et al. (2002), for example, shows some samples grouped and labeled as “Colombian,” rather than as a specific population within the geographic boundaries of the country Colombia. Such difference is due, most likely, to loss of meta-data about the samples, making cross-group comparison in terms of population names problematic.

26 Laurent Excoffier was one of the authors of the Cann et al. (2002) paper announcing the availability of a diversity cell line panel from the HGDP, also in *Science*. This is relevant from an RSK point of view because it shows the complexity of researchers’ stakes, re-alignment and mobility within and among research programs and projects related to a topic.

27 Yet, we don’t include this technical exchange as part of the larger skirmish of three papers because the technical exchange is not the specific focus of the challenge and response papers. Also, including them as part of a 5-paper skirmish would not substantially change our argument or findings about the character of re-situations of objects of knowledge in skirmishes.

28 Marcus W. Feldman was a colleague of Cavalli-Sforza and is the successor to leadership of the Stanford efforts to investigate human evolutionary population genetics. Noah A. Rosenberg and Jonathan K. Pritchard are both former graduate students from Feldman’s laboratory. For an intellectual biography of Cavalli-Sforza including the place of his work on this topic in the larger frame of his research agenda, see Stone and Lurquin (2005).

29 It is yet another issue arising in the literature on HPGA studies how clades relate to the clusters and clines discovered by the approaches described here. Serre and Pääbo write about “continental clades,” yet if we think about the ancestral humans that migrated out of Africa, they presumably formed one monophyletic clade with Africans, while the groups clustering in particular “continental” regions derived from those migrations might not be monophyletic. They may still count as para- or poly-phyletic (sub)clades (on some notions of cladistic analysis), depending on the patterns of branching migrations, back-migrations into Africa, and various other complex migration histories subsequently. We are unsure what Serre and Pääbo meant by “continental clade” in 2004.

30 “Led” in the sense that Rosenberg is first author. How the team was organized and operated to perform the project will be addressed elsewhere (Griesemer & Barragán, 2018).

31 The next year, Rosenberg (2006) proposed a strategy for tracking changes in the datasets used to contribute to HPGA studies and, in particular, those datasets involved in his own work and what consequences the changes in dataset objects of knowledge have for subsequent research work (See Griesemer 2020).

32 As Roberts (1992) reported, there was controversy at the start among proponents of the HGDP whether sampling should be based on recognized “populations” (as Cavalli-Sforza had favored) or on an “objective” sampling grid (as Wilson had favored). Pääbo had been a postdoc with Wilson at UC Berkeley from 1987 to 1990 (https://www.eva.mpg.de/genetics/staff/paabo.html, retrieved April 23, 2021). As a proponent of “grid sampling” rather than “population based” sampling, it is perhaps not surprising that as Wilson’s postdoc, Pääbo aligned on the side of homogeneous geographic sampling designs rather than population based sampling designs.

33 Based on a PUBMED search June 17, 2021. This of course does not mean that the issues posed in the skirmish did not travel to other contexts, as there are several hundred citations of the Serre and Pääbo paper since 2004. It is beyond the scope of our article on this skirmish to trace these broader citation patterns and potential re-situations.

34 Wilson, a biochemist at UC Berkeley, was involved in bringing molecular biology to anthropology, working with anthropologist Vincent Sarich (1934–2012) with microcomplement fixation techniques, using immunological cross-reactivity as a measure of “immunological distance” for inferring relatedness before mitochondrial DNA for inferring phylogeny, also pioneered by Wilson, became available; Sarich and Wilson (1967), see also Footnote 29 in this article, and DiMarco (2020).

35 We acknowledge that some critiques have been co-authored with life-scientists “inside” the specialty, e.g., Fujimura et al. (2014) includes Richard Lewontin as a co-author, as well as population-genetics savvy sociologists and anthropologists, such as Jonathan Marks. However, in this case their critique is directed at a social science paper, Shiao et al. (2012), interpreting the Rosenberg et al. (2002) findings rather than Rosenberg et al. per se. The role(s) of population geneticists in such critiques requires further RSK investigation than we can offer here (see also Sect. 1, and Footnote 20).
